# Association between serum creatinine to albumin ratio and short- and long-term all-cause mortality in patients with acute pancreatitis admitted to the intensive care unit: a retrospective analysis based on the MIMIC-IV database

**DOI:** 10.3389/fimmu.2024.1373371

**Published:** 2024-04-15

**Authors:** Jianjun Wang, Han Li, Huiwen Luo, Ruizi Shi, Sirui Chen, Junchao Hu, Hua Luo, Pei Yang, Xianfu Cai, Yaodong Wang, Xintao Zeng, Decai Wang

**Affiliations:** ^1^Department of Hepatobiliary Surgery, Mianyang Central Hospital, School of Medicine, University of Electronic Science and Technology of China, Mianyang, China; ^2^National Health Commission (NHC) Key Laboratory of Nuclear Technology Medical Transformation, Mianyang Central Hospital, School of Medicine, University of Electronic Science and Technology of China, Mianyang, China; ^3^Department of Cardiology, The Fifth Hospital of Wuhan, Wuhan, China; ^4^Department of Urology, Mianyang Central Hospital, School of Medicine, University of Electronic Science and Technology of China, Mianyang, China

**Keywords:** creatinine to albumin ratio, acute pancreatitis, all-cause mortality, MIMIC-IV, serum biomarker

## Abstract

**Background:**

Serum creatinine (Cr) and albumin (Alb) are important predictors of mortality in individuals with various diseases, including acute pancreatitis (AP). However, most previous studies have only examined the relationship between single Cr or Alb levels and the prognosis of patients with AP. To our knowledge, the association between short- and long-term all-cause mortality in patients with AP and the blood creatinine to albumin ratio (CAR) has not been investigated. Therefore, this study aimed to evaluate the short- and long-term relationships between CAR and all-cause mortality in patients with AP.

**Methods:**

We conducted a retrospective study utilizing data from the Medical Information Market for Intensive Care (MIMIC-IV) database. The study involved analyzing various mortality variables and obtaining CAR values at the time of admission. The X-tile software was used to determine the optimal threshold for the CAR. Kaplan-Meier (K-M) survival curves and multivariate Cox proportional hazards regression models were used to assess the relationship between CAR and both short- and long-term all-cause mortality. The predictive power, sensitivity, specificity, and area under the curve (AUC) of CAR for short- and long-term mortality in patients with AP after hospital admission were investigated using Receiver Operating Characteristic analysis. Additionally, subgroup analyses were conducted.

**Results:**

A total of 520 participants were included in this study. The CAR ideal threshold, determined by X-tile software, was 0.446. The Cox proportional hazards model revealed an independent association between CAR≥0.446 and all-cause mortality at 7-day (d), 14-d, 21-d, 28-d, 90-d, and 1-year (y) before and after adjustment for confounders. K-M survival curves showed that patients with CAR≥0.446 had lower survival rates at 7-d, 14-d, 21-d, 28-d, 90-d, and 1-y. Additionally, CAR demonstrated superior performance, with higher AUC values than Cr, Alb, serum total calcium, Glasgow Coma Scale, Systemic Inflammatory Response Syndrome score, and Sepsis-related Organ Failure Assessment score at 7-d, 14-d, 21-d, 28-d, 90-d, and 1-y intervals. Subgroup analyses showed that CAR did not interact with a majority of subgroups.

**Conclusion:**

The CAR can serve as an independent predictor for short- and long-term all-cause mortality in patients with AP. This study enhances our understanding of the association between serum-based biomarkers and the prognosis of patients with AP.

## 1 Introduction

Acute pancreatitis (AP) is a prevalent disease of the digestive system, affecting 13-45 per 100,000 individuals annually, with gallstones, alcoholism, and triglyceridemia being the main causes (1, 2). Most patients are mildly symptomatic, presenting with epigastric or full abdominal pain, abdominal distension and vomiting, which can be controlled with supportive care and intravenous fluid therapy (3, 4). Approximately 20% of patients develop moderate or severe AP, with pancreatic necrosis and peripancreatic tissue necrosis, and even multi-organ failure, with a mortality rate of up to 30% in this group of patients (5–7). Therefore, early and accurate assessment of disease severity may help to reduce AP mortality.

The Ranson criteria (8), Balthazar grade (9), Sepsis-related Organ Failure Assessment (SOFA) (10), Acute Physiology and Chronic Health Evaluation II (APAChE-II) (8), and Bedside Index for Severity in AP (BISAP) (11) are now the most widely used approaches for assessing AP severity. However, these scoring systems are complex. Examples include the Ranson criteria, which includes 11 clinical and laboratory data, and APAChE-II, which includes age, chronic health status, and 12 acute physiological markers. Consequently, these scoring systems require time for data collection before concluding the condition assessment, which may cause a delay in determining the severity of the disease and miss the optimal window of opportunity for optimal treatment. Therefore, there is an urgent need for a simpler, quicker, highly repeatable, and sensitive indicator to gauge AP severity.

Previous studies have demonstrated a relationship between blood levels of serum creatinine (Cr), albumin (Alb), and C-reactive protein (CRP) with the severity and prognosis of AP (12–14). Combining these serum indicators, despite having low predictive value, effectively enhances predictive sensitivity (15). CRP and Cr levels are usually elevated in patients with AP; however, albumin levels frequently show varying degrees of decline. Previous studies have shown that CRP/Alb is a valuable tool for predicting the probability of death in patients with AP (16). According to Kaplan et al, in patients with AP, the risk of death increases 1.52-fold for every unit rise in CRP/Alb (16). Moreover, a CRP/Alb ratio >16.28, with 92.1% sensitivity and 58.0% specificity, is a significant predictor of mortality (16). Additionally, there is a strong link between Cr levels, prognosis, and the degree of AP (13). According to research by Lankisch et al. (13), blood Cr levels (≥ 2 mg/dl) upon admission, as well as 24 and 48 hours later, were useful in determining if the pancreas was necrotic. The creatinine-to-albumin ratio (CAR), according to Zhao et al. (17), is a practical, consistent, and easy-to-measure biochemical tool used to evaluate the effectiveness of pancreatitis debridement surgery.

However, the relationship between CAR and short- and long-term all-cause mortality in patients with AP remains unclear. Thus, we used the Medical Information Mart for Intensive Care IV version 2.2 [MIMIC-IV (v2.2)] database to collect data on the hospitalization status of patients with AP admitted between 2008 and 2019. The study aimed to examine the association between short- and long-term all-cause mortality and CAR in these patients.

## 2 Methods

### 2.1 Data source

The MIMIC-IV (v2.2) database, a large, openly available database created by the Massachusetts Institute of Technology’s Laboratory of Computational Physiology, provided all data used in this investigation (18). The official link is https://mimic.mit.edu/. MIMIC-IV is an invaluable resource for research in clinical decision support, predictive modeling, critical care outcomes, and related fields, owing to its extensive collection of Intensive Care Unit (ICU)-specific data. All patients admitted to the Beth Israel Deaconess Medical Center between 2008 and 2019 were included. All test results, prescription schedules, vital signs, length of hospital stay for each patient, and other specific information were recorded. To protect patient privacy, all personal data were de-identified, and patient identification was substituted with random numbers. Ethical approval and informed consent were waived.

### 2.2 Criteria for population selection and results

**Figure 1:**
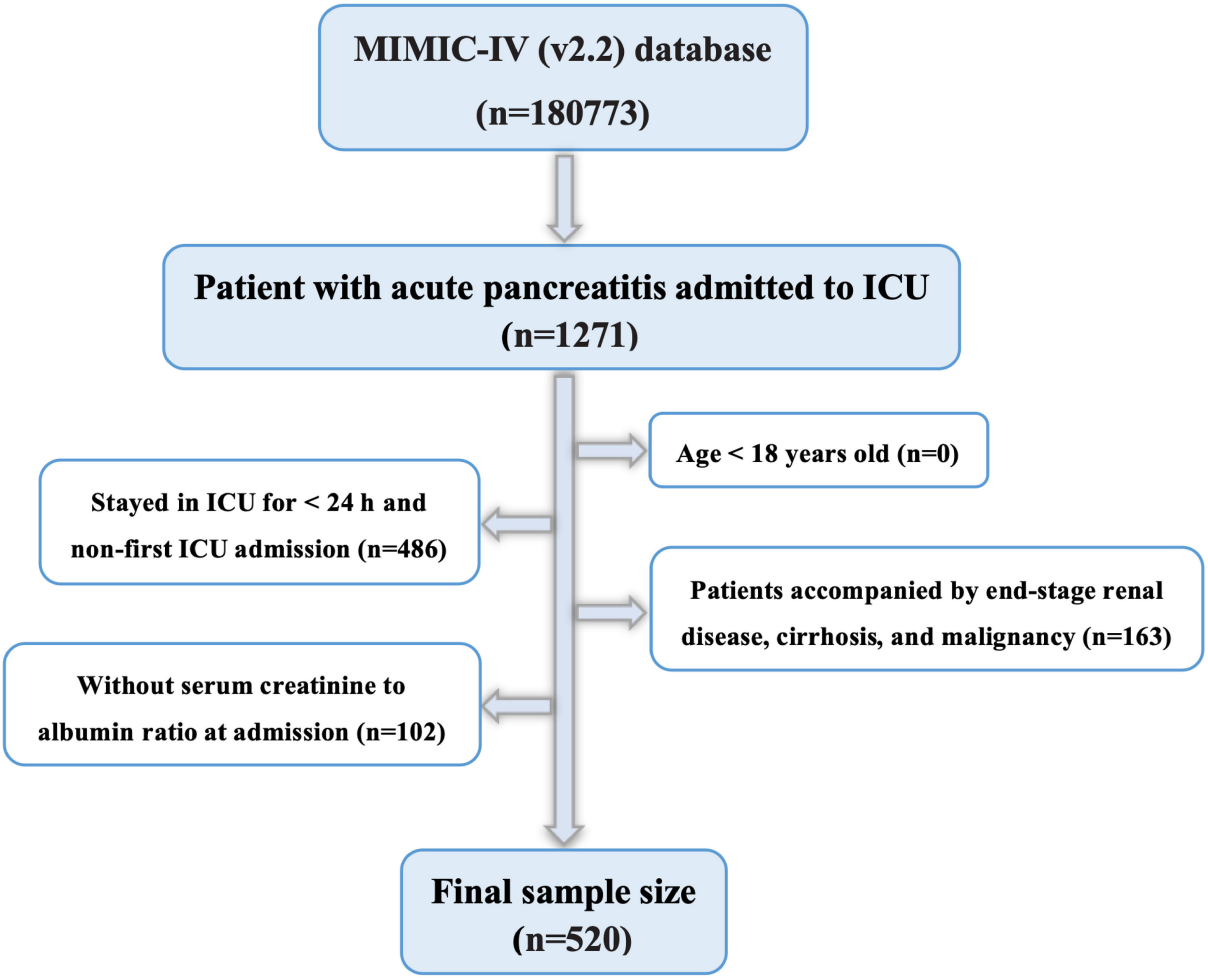
Flowchart for participants from the MIMIC-IV (v 2.2).

The MIMIC-IV database contains data on 180,733 individuals admitted to the ICU between 2012 and 2019. Hospital admission data for patients with AP were retrieved using the International Classification of Diseases, 9th Revision (ICD-9) code 577.0, and International Classification of Diseases, 10th Revision (ICD-10) codes K85-K85.92. A total of 5,894 patients with AP were included, of whom 1,271 were admitted to the ICU. Data regarding the initial ICU admissions of patients aged 18 years or older were collected for this study. The following patients were also excluded from this study: those who had end-stage renal disease, cirrhosis, malignancy, or recurrent admissions for acute pancreatitis, retaining only the initial admission data for this subgroup; those who spent less than 24 hours in the ICU; and those for whom Cr and Alb data were not recorded within 24 hours of admission. Finally, 520 patients were included in the study (**Figure 1**).

### 2.3 Data extraction

CAR was chosen as the main study variable. Serum Cr and Alb levels were examined for the first time after admission to minimize treatment-related interference. All variables were extracted from the MIMIC-IV database using a Structured Query Language (SQL) with PostgreSQL. Demographic information, vital signs, clinical treatments, comorbidities, laboratory data, and treatment outcomes were the six main components of the extraction procedure. **Table 1** presents a comprehensive list of the extracted variables.

**Table 1:** Covariates extracted in detail from the MIMIC-IV database.

Items	Composition
Demographic variables	Age, Gender, Race
Vital Signs	HR, SBP, DBP, MAP, RR, SpO2, Temperature
Clinical Treatments	Vasopressin, Octreotide, Statins, Betablockers, MV, CRRT, ERCP
Comorbidities	AKI, Sepsis, RF, HF, AF, Hypertension, Diabetes, Obesity
Laboratory variables	Neutrophil counts, Nymphocyte counts, Eosinophil counts, RBC, WBC, RDW, Plt, Hb, Lymphocyte percentage, HCT, Cr, Alb, BUN, TB, AST, ALT, Glucose, TC, TG, HDL-C, LDL-C, PT, INR, Blood amylase, Blood lipase, K, Na, TCa, AG, Lac
Clinical Outcomes	LOS ICU, LOS hospital, ICU mortality, In-hospital mortality, 7-day mortality, 14-day mortality, 21-day mortality, 28-day mortality, 90-day mortality, 1-year mortality

MIMIC-IV, the Medical Information Mart for Intensive Care database; HR, heart rate; SBP, systolic blood pressure; DBP, diastolic blood pressure; MAP, mean arterial pressure; RR, respiratory rate; MV, mechanical ventilation; CRRT, continuous renal replacement therapy; ERCP, endoscopic retrograde cholangiopancreatography; AKI, acute kidney injury; RF, respiratory failure; HF, heart failure; AF, atrial fibrillation; RBC, red blood cell; WBC, white blood cell; RDW, erythrocyte distribution width; Plt, platelet; Hb, hemoglobin; HCT, hematocrit; Cr, creatinine; Alb, albumin; BUN, blood urea nitrogen; TB, total bilirubin; AST, aspartate aminotransferase; ALT, alanine aminotransferase; TC, total cholesterol; TG, triglyceride; HDL-C, high-density lipoprotein cholesterol; LDL-C, low-density lipoprotein cholesterol; PT, prothrombin time; INR, international normalized ratio; K, serum potassium; Na, serum sodium; TCa, serum total calcium; AG, anion gap; Lac, lactate; CAR, serum creatinine to albumin ratio; LOS ICU, length of ICU stay; LOS hospital, length of hospital stay.

### 2.4 Endpoint events

The primary endpoint of the study was all-cause mortality within 7-d, 14-d, 21-d, 28-d, 90-d, and 1-y of admission after hospital admission. Additionally, we also assessed the duration of stay in the ICU, the total length of stay, as well as ICU and hospital mortality during this period.

### 2.5 Statistical analysis

**Figure 2:**
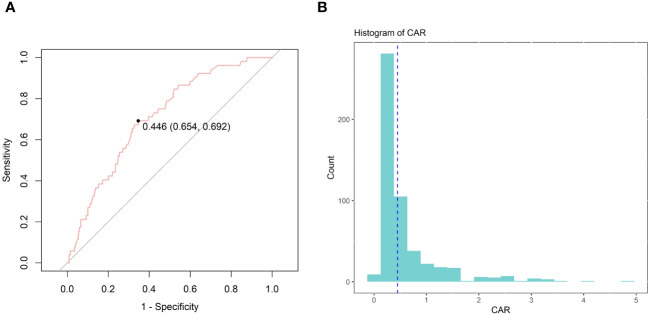
The choice of the optimal cutoff point maximized the risk ratio **(A)** and the relationship between CAR≥0.446 and the distribution of CAR **(B)**.

Continuous variables were expressed as mean ± standard deviation (SD) for normally distributed variables, median (IQR) for non-normally distributed continuous variables, and numbers (%) for categorical variables. The T-test or One-Way ANOVA was used to compare continuous variables, while Pearson’s χ^2^ test and Fisher’s test were used to compare categorical variables when examining baseline characteristics. The X-tile software (Version 3.6.1, Yale University School of Medicine) is commonly used to calculate optimal thresholds in survival analyses and continuous data from medical or epidemiologic studies. After determining the ideal CAR cutoff value based on whether death occurred on day 28 after admission, we divided the study patient population into two groups: low CAR and high CAR. The choice of the optimal cutoff point that maximized the risk ratio is presented in **Figure 2**, as well as the relationship of CAR≥0.446 and the distribution of CAR. Univariate and multivariate analyses of prognostic indicators were performed using the Cox proportional hazards model to identify independent predictors of death in patients with AP 7-d, 14-d, 21-d, 28-d, 90-d, and 1-y after admission. HRs with 95% confidence intervals (CIs) are provided. Unadjusted survival curves were drawn using the Kaplan-Meier (K-M) method, and a log-rank test was used to compare the two sets of curves. The predictive power of CAR, Cr, Alb, and SOFA scores for mortality at 7-d, 14-d, 21-d, 28-d, 90-d, and 1-y after admission was assessed using Receiver Operating Characteristic (ROC) analysis. The sensitivity and specificity for each indicator were determined, and the area under the curve (AUC) was calculated. Subgroup analyses were conducted to examine the potential impact of CAR on various parameters, such as age, sex, acute kidney injury (AKI), hypertension, and diabetes. The R statistical program (R version 4.2.2, R Foundation for Statistical Computing), SPSS Statistics 26 (IBM, Chicago, IL, USA), and GraphPad Prism 8 (GraphPad Software, San Diego, CA, USA) were the software programs utilized for the studies.

## 3 Results

### 3.1 Baseline demographic and clinical characteristics

Ultimately, 520 patients with AP receiving treatment in the ICU were included in this study (**Figure 1**). Notably, 231 (44.4%) were women, and 289 were men (55.6%). Using X-tile software, the best CAR cutoff was determined based on whether the patient died on day 28 following admission. The results showed that 0.446 was the ideal CAR threshold (**Figure 2**). The study population was divided into two groups: those with a low CAR (<0.446) and those with a high CAR (≥ 0.446) and those with the high CAR group had lower mean arterial pressure, systolic and diastolic blood pressure, and body temperature, and greater rates of vasopressin and continuous renal replacement therapy. In addition, patients with a combination of AKI, sepsis, and respiratory failure (RF) were more likely to be in the high CAR group. Among the laboratory markers, patients in the high CAR group had elevated levels of lactate, anion gap, serum potassium, international normalized ratio, blood urea nitrogen, prothrombin time, and creatinine. Subsequent research revealed a greater risk of poor prognosis for patients in the high CAR group. They had higher ICU mortality (4.0% vs. 13.1%, *P* < 0.001), hospitalization mortality (6.2% vs. 19.2%, *P* < 0.001), 7-d mortality (1.6% vs. 6.1%, *P* = 0.011), 14-d mortality (3.7% vs. 12.1%, *P* < 0.001), 21-d mortality (4.7% vs. 16.2%, *P* < 0.001), 28-d mortality (5.0% vs. 18.2%, *P* < 0.001), 90-d mortality (10.2% vs. 27.8%, *P* < 0.001) and 1-y mortality (14.3% vs. 33.3%, *P* < 0.001). Furthermore, the high CAR group had longer ICU hospitalizations [(3.18 (1.86, 6.66) vs. 5.84 (2.37, 13.16), *P* < 0.001)] and longer hospital stays overall [11.33 (6.96, 21.34) vs. 16.71 (9.90, 28.76), *P* < 0.001)]. The detailed results are presented in **Table 2**.

**Table 2:** Baseline patient characteristics in patients with acute pancreatitis.

Variable	Total (n=520)	CAR		*P* value
		<0.446 (n=322)	≥0.446 (n=198)	
Demographics				
Age	59.00 (46.00, 74.00)	55.00 (43.00, 70.00)	65.00 (53.25, 76.00)	<0.001
Gender				0.057
Male	289 (55.6)	168 (52.2)	121 (61.1)	
Female	231 (44.4)	154 (47.8)	77 (38.9)	
Race, n (%)				0.399
Asian	20 (3.8)	9 (2.8)	11 (5.6)	
White	324 (62.3)	205 (63.7)	119 (60.1)	
Black	38 (7.3)	22 (6.8)	16 (8.1)	
Other	138 (26.5)	86 (26.7)	52 (26.3)	
Vital Signs				
HR	100.00 (84.00, 116.00)	101.00 (85.00, 116.75)	99.00 (83.00, 115.00)	0.356
SBP	127.00 (109.75, 144.25)	131.00 (114.00, 150.75)	120.50 (101.00, 140.00)	<0.001
DBP	72.00 (60.00, 85.00)	75.00 (63.00, 88.00)	66.00 (56.00, 80.00)	<0.001
MAP	90.67 (78.00, 103.83)	94.33 (80.42, 107.00)	85.33 (73.42, 97.83)	<0.001
Respiratory rate	21.00 (17.00, 26.00)	21.00 (18.00, 25.00)	21.00 (17.00, 26.00)	0.988
Spo2	96.00 (94.00, 99.00)	95.88 (94.00, 99.00)	96.00 (94.00, 98.00)	0.255
Temperature	36.89 (36.44, 37.39)	36.94 (36.56, 37.56)	36.67 (36.34, 37.22)	<0.001
Clinical Treatment				
Vasopressin	79 (15.2)	23 (7.1)	56 (28.3)	<0.001
Octreotide	29 (5.6)	17 (5.3)	12 (6.1)	0.857
Statins	171 (32.9)	99 (30.7)	72 (36.4)	0.219
Betablockers	308 (59.2)	179 (55.6)	129 (65.2)	0.039
MV	460 (88.5)	280 (87.0)	180 (90.9)	0.219
CRRT	61 (11.7)	10 (3.1)	51 (25.8)	<0.001
ERCP	31 (6.0)	26 (8.1)	5 (2.5)	0.016
Comorbidities				
AKI	360 (69.2)	195 (60.6)	165 (83.3)	<0.001
Sepsis	381 (73.3)	209 (64.9)	172 (86.9)	<0.001
RF	234 (45.0)	117 (36.3)	117 (59.1)	<0.001
HF	93 (17.9)	52 (16.1)	41 (20.7)	0.230
AF	115 (22.1)	65 (20.2)	50 (25.3)	0.214
Hypertension	252 (48.5)	181 (56.2)	71 (35.9)	<0.001
Diabetes	164 (31.5)	95 (29.5)	69 (34.8)	0.239
Obesity	69 (13.3)	41 (12.7)	28 (14.1)	0.744
Laboratory Indicators				
Neutrophil counts	9.08 (5.27, 14.52)	8.41 (4.92, 14.27)	9.99 (6.46, 15.55)	0.053
Nymphocyte counts	1.03 (0.59, 1.73)	1.11 (0.63, 1.73)	0.90 (0.55, 1.76)	0.378
Eosinophil counts	0.01 (0.00, 0.11)	0.01 (0.00, 0.10)	0.01 (0.00, 0.12)	0.714
RBC	3.71 (3.20, 4.23)	3.76 (3.25, 4.22)	3.59 (3.13, 4.23)	0.256
WBC	13.50 (9.40, 18.70)	13.30 (9.35, 18.55)	13.80 (9.43, 19.17)	0.656
RDW	14.50 (13.60, 15.70)	14.30 (13.50, 15.50)	14.70 (13.93, 15.97)	<0.001
PLT	194.00 (135.00, 275.00)	205.00 (146.00, 287.00)	176.50 (125.00, 239.50)	0.002
Hb	11.30 (9.70, 13.00)	11.40 (9.90, 12.93)	11.00 (9.60, 13.17)	0.226
Lymphocyte percentage	8.00 (4.00, 13.57)	8.10 (4.32, 14.20)	6.85 (3.68, 12.00)	0.011
HCT	34.00 (29.60, 38.80)	34.30 (29.98, 38.40)	33.30 (29.40, 39.50)	0.744
Cr	1.05 (0.70, 1.70)	0.80 (0.60, 1.00)	2.05 (1.50, 3.40)	<0.001
Alb	2.90 (2.50, 3.30)	3.00 (2.62, 3.40)	2.70 (2.30, 3.18)	<0.001
BUN	20.00 (12.00, 33.00)	15.00 (10.00, 20.00)	37.50 (25.25, 56.00)	<0.001
TB	1.00 (0.60, 2.60)	0.90 (0.60, 2.32)	1.10 (0.60, 2.90)	0.672
AST	62.00 (33.00, 158.00)	57.00 (32.00, 149.50)	71.00 (38.00, 181.00)	0.088
ALT	54.00 (24.00, 152.00)	51.00 (24.00, 141.00)	59.00 (24.75, 160.00)	0.523
Glucose	134.00 (105.00, 174.50)	130.00 (106.00, 164.75)	137.00 (105.00, 188.00)	0.105
TC	155.50 (116.00, 199.50)	170.00 (127.75, 206.25)	139.50 (107.00, 181.25)	0.008
TG	183.00 (107.00, 326.00)	163.50 (103.00, 293.75)	201.00 (126.00, 366.00)	0.101
HDL-C	26.00 (14.75, 35.25)	30.00 (23.00, 38.00)	21.00 (12.00, 32.00)	0.018
LDL-C	58.50 (38.75, 89.75)	66.00 (38.75, 98.25)	52.00 (40.50, 84.25)	0.317
PT	14.30 (12.90, 16.20)	14.10 (12.70, 15.70)	14.90 (13.00, 17.50)	<0.001
INR	1.30 (1.20, 1.50)	1.30 (1.10, 1.40)	1.30 (1.20, 1.60)	<0.001
Blood amylase	131.00 (53.00, 375.75)	101.00 (50.00, 285.00)	176.00 (63.00, 482.00)	0.008
Blood lipase	223.00 (61.00, 1222.00)	166.00 (50.50, 1010.00)	356.50 (87.25, 1522.00)	0.001
K	4.00 (3.60, 4.60)	3.90 (3.60, 4.30)	4.30 (3.82, 4.90)	<0.001
Na	138.00 (135.00, 142.00)	138.00 (136.00, 141.00)	139.00 (135.00, 142.75)	0.199
TCa	7.90 (7.30, 8.40)	8.00 (7.40, 8.50)	7.70 (7.00, 8.28)	<0.001
AG	14.00 (12.00, 16.00)	13.00 (11.00, 15.00)	15.00 (12.00, 18.00)	<0.001
Lac	1.70 (1.20, 2.70)	1.60 (1.10, 2.30)	2.10 (1.30, 3.20)	<0.001
CAR	0.35 (0.25, 0.60)	0.27 (0.21, 0.33)	0.78 (0.53, 1.33)	<0.001
Clinical Outcomes				
LOS ICU	3.77 (1.96, 8.98)	3.18 (1.86, 6.66)	5.84 (2.37, 13.16)	<0.001
LOS hospital	13.71 (7.71, 23.76)	11.33 (6.96, 21.34)	16.71 (9.90, 28.76)	<0.001
Hospital mortality	58 (11.2)	20 (6.2)	38 (19.2)	<0.001
ICU mortality	39 (7.5)	13 (4.0)	26 (13.1)	<0.001
7-day mortality	17 (3.3)	5 (1.6)	12 (6.1)	0.011
14-day mortality	36 (6.9)	12 (3.7)	24 (12.1)	<0.001
21-day mortality	47 (9.0)	15 (4.7)	32 (16.2)	<0.001
28-day mortality	52 (10.0)	16 (5.0)	36 (18.2)	<0.001
90-day mortality	88 (16.9)	33 (10.2)	55 (27.8)	<0.001
1-year mortality	112 (21.5)	46 (14.3)	66 (33.3)	<0.001

HR, heart rate; SBP, systolic blood pressure; DBP, diastolic blood pressure; MAP, mean arterial pressure; RR, respiratory rate; MV, mechanical ventilation; CRRT, continuous renal replacement therapy; ERCP, endoscopic retrograde cholangiopancreatography; AKI, acute kidney injury; RF, respiratory failure; HF, heart failure; AF, atrial fibrillation; RBC, red blood cell; WBC, white blood cell; RDW, erythrocyte distribution width; Plt, platelet; Hb, hemoglobin; HCT, hematocrit; Cr, creatinine; Alb, albumin; BUN, blood urea nitrogen; TB, total bilirubin; AST, aspartate aminotransferase; ALT, alanine aminotransferase; TC, total cholesterol; TG, triglyceride; HDL-C, high-density lipoprotein cholesterol; LDL-C, low-density lipoprotein cholesterol; PT, prothrombin time; INR, international normalized ratio; K, serum potassium; Na, serum sodium; TCa, serum total calcium; AG, anion gap; Lac, lactate; CAR, serum creatinine to albumin ratio; LOS ICU, length of ICU stay; LOS hospital, length of hospital stay.

### 3.2 Univariate and multivariate Cox regression models of CAR with mortality in patients with AP

Univariate and multivariate Cox regression analyses were conducted to identify the potential relationship between the CAR and mortality in patients with AP. Elevated CAR (≥0.446) was substantially linked to an increased risk of mortality at various periods in the unadjusted initial model: 7-d (HR = 3.98, 95% CI: 1.40-11.28, *P* for trend = 0.010), 14-d (HR = 3.40, 95% CI: 1.70-6.80, *P* for trend = 0.001), 21-d (HR = 3.68, 95% CI: 1.99-6.79, *P* for trend < 0.001), 28-d (HR = 3.91, 95% CI: 2.17-7.05, *P* for trend < 0.001), 90-d (HR = 3.04, 95% CI: 1.97-4.68, *P* for trend < 0.001) and 1-y (HR = 2.70, 95% CI: 1.85-3.93, *P* for trend < 0.001). In the multivariate Model 1, the group of patients with CAR≥0.446 maintained a higher risk of mortality even after adjusting for confounding factors, including age and sex: 7-d (HR = 3.82, 95% CI: 1.33-10.94, *P* for trend = 0.013), 14-d (HR = 2.99, 95% CI: 1.48-6.04, *P* for trend = 0.002), 21-d (HR = 3.11, 95% CI: 1.67-5.81, *P* for trend < 0.001), 28-d (HR = 3.39, 95% CI: 1.86-6.16, *P* for trend < 0.001), 90-d (HR = 2.67, 95% CI: 1.72-4.15, *P* for trend < 0.001) and 1-y (HR = 2.33, 95% CI: 1.59-3.43, *P* for trend < 0.001). A subsequent multivariate model (Model 2) that included additional possible confounders also revealed an independent correlation between a higher CAR and an increased likelihood of mortality during the previously mentioned periods. The detailed results are presented in **Table 3**.

**Table 3:** Univariate and multivariate Cox regression models of CAR with mortality in patients with AP.

Outcome	Unadjusted, HR (95%CI)	Model 1, HR (95%CI)	Model 2, HR (95%CI)
7-d			
CAR<0.446	1	1	1
CAR≥0.446	3.98 (1.40-11.28)	3.82 (1.33-10.94)	3.63 (1.19-11.04)
*P* for trend	0.010	0.013	0.023
14-d			
CAR<0.446	1	1	1
CAR≥0.446	3.40 (1.70-6.80)	2.99 (1.48-6.04)	3.11 (1.52-6.36)
*P* for trend	0.001	0.002	0.002
21-d			
CAR<0.446	1	1	1
CAR≥0.446	3.68 (1.99-6.79)	3.11 (1.67-5.81)	3.34 (1.77-6.30)
*P* for trend	<0.001	<0.001	<0.001
28-d			
CAR<0.446	1	1	1
CAR≥0.446	3.91 (2.17-7.05)	3.39 (1.86-6.16)	3.58 (1.95-6.59)
*P* for trend	<0.001	<0.001	<0.001
90-d			
CAR<0.446	1	1	1
CAR≥0.446	3.04 (1.97-4.68)	2.67 (1.72-4.15)	2.77 (1.77-4.34)
*P* for trend	<0.001	<0.001	<0.001
1-year			
CAR<0.446	1	1	1
CAR≥0.446	2.70 (1.85-3.93)	2.33 (1.59-3.43)	2.39 (1.61-3.54)
*P* for trend	<0.001	<0.001	<0.001

Model 1: Adjusted age and gender.

Model 2: Model 1+ Spo2+Temperature+Vasopressin+Betablockers+Plt+HCT.

### 3.3 Kaplan-Meier curve and ROC curve analysis

**Figure 3:**
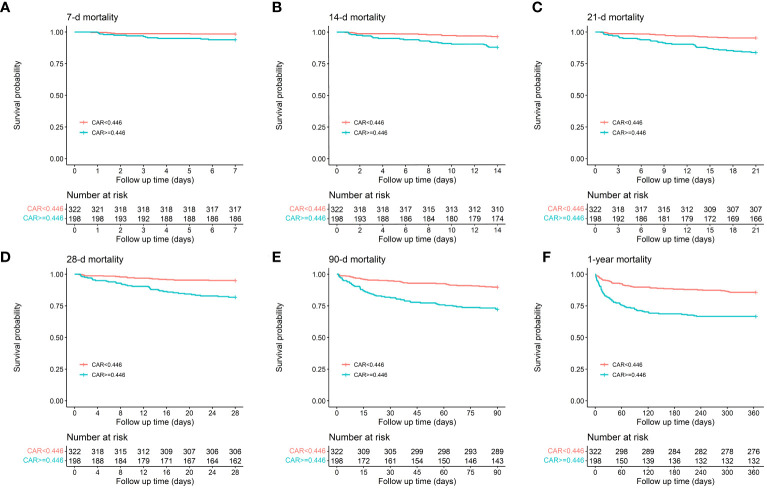
Kaplan-Meier survival analysis curves for all-cause mortality in patients with AP at 7-d **(A)**, 14-d **(B)**, 21-d **(C)**, 28-d **(D)**, 90-d **(E)**, and 1-y **(F)** of hospital admission.

Patients with CAR≥0.446 had higher mortality at 7-d, 14-d, 21-d, 28-d, 90-d, and 1-y intervals compared to those with CAR < 0.446, as demonstrated by the K-M survival curves (1.6% vs. 6.1%, *P* = 0.011; 3.7% vs. 12.1%, *P* < 0.001; 4.7% vs. 16.2%, *P* < 0.001; 5.0% vs. 18.2%, *P* < 0.001; 10.2% vs. 27.8%, *P* < 0.001; 14.3% vs. 33.3%, *P* < 0.001). Patients with CAR levels≥0.446 also had increased ICU and hospital mortality rates (4.0% vs. 13.1%, *P* < 0.001; 6.2% vs. 19.2%, *P* < 0.001). The results are presented in **Figure 3**.

**Figure 4:**
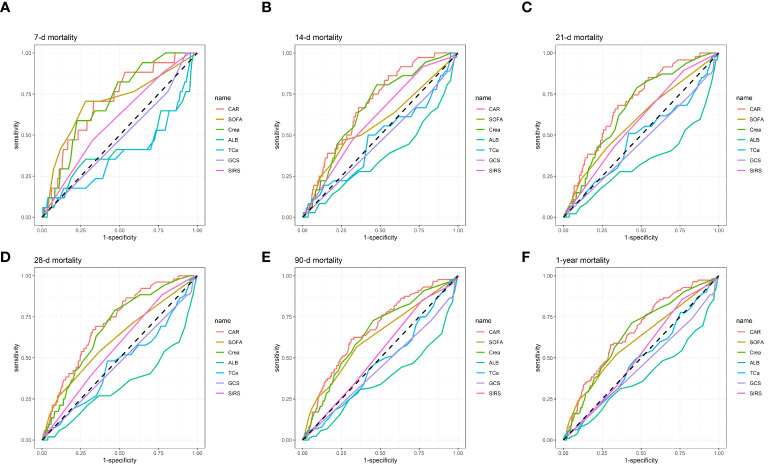
ROC curves for predicting all-cause mortality in patients with AP at 7-d **(A)**, 14-d **(B)**, 21-d **(C)**, 28-d **(D)**, 90-d **(E)**, and 1-y **(F)** after admission.

ROC curves were generated for predicting all-cause death in patients at 7-d, 14-d, 21-d, 28-d, 90-d, and 1-y following admission using seven measures: Cr, Alb, serum total calcium (TCa), Glasgow Coma Scale (CGS), Systemic Inflammatory Response Syndrome (SIRS) score, and SOFA score (**Figure 4**). For specific details regarding **Figure 4**, please refer to **Table 4**. Our results revealed that, in terms of AUC values, CAR was superior to Cr, Alb, TCa, CGS, SIRS score, and SOFA score at 7-d, 14-d, 21-d, 28-d, 90-d, and 1-y intervals. For example, compared to Cr [67.91% (95% CI: 60.98%-74.84%)], Alb [34.08% (95% CI: 25.34%-42.83%)], TCa [46.59% (95% CI: 37.99-55.19)], CGS [47.43% (95% CI: 41.21-53.65)], SIRS [56.89% (95% CI: 49.74-64.04)], and SOFA [61.06% (95% CI: 52.68%-69.44%)], CAR had a considerably improved AUC on day 28 of admission [70.98% (95% CI: 64.26%-77.69%)]. Similarly, the AUC for CAR was considerably higher on day 90 of admission [67.23% (95% CI: 61.17%-73.30%)] than for Alb [36.33% (95% CI: 29.41%-43.24%), Cr [64.77% (95% CI: 58.53%-71.01%), TCa [46.81% (95% CI: 40.21-53.41)], CGS [44.56% (95% CI: 39.39-49.72)], SIRS [52.78% (95% CI: 46.85-58.70)], and SOFA [62.47% (95% CI: 55.91%-69.03%)]. The detailed results are presented in **Table 4**. Consequently, our findings highlight the significant predictive benefits of the CAR.

**Table 4:** Information of ROC curves in **Figure 4**.

Variables	AUC (%)	95%CI (%)	Threshold	Sensitivity	Specificity
7-d					
CAR	70.68	58.77-82.59	0.486	0.706	0.668
SOFA	69.90	55.35-84.44	2.500	0.706	0.720
Cr	70.49	59.81-81.18	1.750	0.588	0.775
Alb	41.54	24.52-58.56	3.250	0.353	0.718
TCa	39.14	23.15-55.12	9.350	0.118	0.962
GCS	48.52	38.69-58.35	13.500	1.000	0.072
SIRS	59.26	46.87-71.64	3.500	0.471	0.672
14-d					
CAR	69.13	61.33-76.94	0.317	0.861	0.446
SOFA	57.19	46.86-67.53	2.500	0.472	0.719
Cr	66.74	58.37-75.11	1.050	0.806	0.523
Alb	37.41	27.01-47.81	1.450	1.000	0.006
TCa	48.13	37.59-58.66	8.050	0.500	0.578
GCS	46.85	39.05-54.12	8.500	1.000	0.019
SIRS	60.51	52.19-68.84	3.500	0.472	0.678
21-d					
CAR	69.65	62.53-76.77	0.446	0.681	0.649
SOFA	59.81	50.53-67.82	2.500	0.426	0.719
Cr	66.71	59.42-74.00	1.050	0.787	0.529
Alb	34.57	25.28-43.86	1.450	1.000	0.006
TCa	48.35	39.14-57.56	8.050	0.511	0.581
GCS	46.07	39.34-52.80	8.500	1.000	0.019
SIRS	58.04	50.57-65.50	2.500	0.894	0.226
28-d					
CAR	70.98	64.26-77.69	0.446	0.692	0.654
SOFA	61.06	52.68-69.44	1.500	0.538	0.630
Cr	67.91	60.98-74.84	1.050	0.788	0.532
Alb	34.08	25.34-42.83	1.450	1.000	0.006
TCa	46.59	37.99-55.19	8.050	0.481	0.578
GCS	47.43	41.21-53.65	8.500	1.000	0.019
SIRS	56.89	49.74-64.04	2.500	0.885	0.226
90-d					
CAR	67.23	61.17-73.30	0.465	0.614	0.688
SOFA	62.47	55.91-69.03	1.500	0.568	0.650
Cr	64.77	58.53-71.01	1.050	0.727	0.546
Alb	36.33	29.41-43.24	1.250	1.000	0.002
TCa	46.81	40.21-53.41	5.800	0.977	0.046
GCS	44.56	39.39-49.72	3.500	0.989	0.012
SIRS	52.78	46.85-58.70	2.500	0.852	0.229
1-year					
CAR	66.39	60.85-71.93	0.465	0.580	0.696
SOFA	60.53	54.53-66.53	1.500	0.527	0.652
Cr	64.65	58.98-70.32	1.050	0.714	0.559
Alb	38.70	32.42-44.99	1.250	1.000	0.002
TCa	48.97	42.92-55.02	7.950	0.491	0.538
GCS	45.32	40.76-49.88	3.500	0.991	0.012
SIRS	52.33	47.01-57.65	2.500	0.857	0.235

ROC, receiver operating characteristic; AUC, area under the curve; CI, confidence interval; CAR, serum creatinine to albumin ratio; Cr, creatinine; Alb, albumin; TCa, serum total calcium; SOFA, Sepsis-related Organ Failure Assessment score; GCS, Glasgow Coma Scale; SIRS, Systemic Inflammatory Response Syndrome score.

### 3.4 Subgroup analyses for the CAR on clinical outcomes in patients with AP

**Figure 5:**
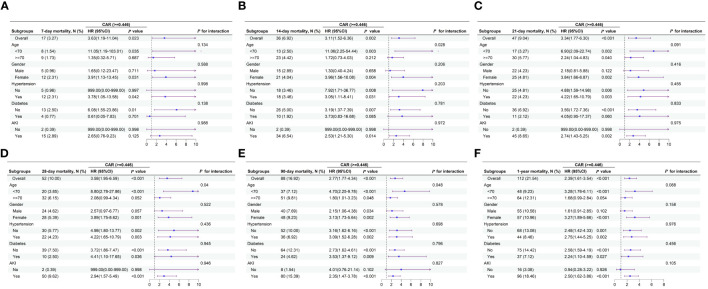
Forest plots of subgroup analysis of the relationship between all-cause mortality and CAR in patients with AP admitted 7-d **(A)**, 14-d **(B)**, 21-d **(C)**, 28-d **(D)**, 90-d **(E)**, and 1-y **(F)**.

**Figure 5** illustrates the presence of the relationship between the 7-d, 14-d, 21-d, 28-d, 90-d, and 1-y CAR and all-cause mortality in different subgroups of patients with AP. When stratified analyses were performed for age, sex, AKI, hypertension, and diabetes, the forest plots showed no significant interaction between CAR and most subgroups (*P* > 0.05), except at 14-d, 28-d, and 90-d, where a small interaction between CAR and age subgroups was observed (*P* for interaction=0.028, 0.04, and 0.048). The results are shown in **Figure 5**.

## 4 Discussion

Numerous recent studies have examined serum biomarkers, such as the neutrophil/lymphocyte ratio (19, 20), red blood cell distribution width/platelet ratio (21), blood glucose/lymphocyte ratio (22), and CRP/Alb ratio (16), to determine the prognosis of AP. However, no research has been published on the use of CAR to predict the prognosis of patients with AP. Our results suggest that among patients with AP, CAR is an independent factor influencing both short- and long-term all-cause mortality. The short- and long-term all-cause mortality was significantly higher in AP patients with CAR≥0.446 than in patients with CAR < 0.446, according to K-M survival analysis plots. Our findings demonstrated that for both short and long-term intervals, CAR outperformed Cr, Alb, TCa, CGS, SIRS score, and SOFA score in terms of AUC values. Subgroup analyses suggested that CAR did not significantly interact with the most of the subgroups.

Numerous studies have demonstrated that elevated Cr is an adverse prognostic indicator of AP (13, 19, 23, 24). Long-established markers of renal function, such as creatinine and blood urea nitrogen (BUN), have variable accuracy in predicting the severity of pancreatitis. Cr is a reasonably priced, easily accessible, and highly dependable blood marker that strongly correlates with renal function. Furthermore, Cr is less sensitive to minor changes in intravascular blood volume and more indicative of internal organ damage (24). Talamini et al. (23) reported that in 192 patients with new-onset pancreatitis, Cr levels > 2 mg/dl were a significant predictor of mortality. Muddana et al. (24) found that elevated Cr was a significant predictor of necrosis in AP based on a study of 185 patients. AKI is a common complication of AP (25, 26). Devani et al. (27) reported that 7.9% of hospitalized patients with AP experienced AKI. Furthermore, older age, male sex, sepsis, RF, and chronic kidney disease (CKD) are associated with an increased risk of concomitant AKI in patients with AP (27).

Nevertheless, the pathophysiologic mechanism behind AKI in individuals with AP remain incompletely understood (28). However, the key pathophysiological mechanism is the early activation of pancreatic enzymes in acinar cells, which results in autodigestion of the pancreas and surrounding tissues (29). This sets off a series of events that ultimately lead to AKI (29). An increase in abdominal pressure, hypovolemia, hypotension, severe vasoconstriction of the renal vasculature, hypercoagulability, and fibrin deposition in the glomeruli are consequences of activated enzymes and proteases released into the circulatory system and damage to the vascular endothelium (30). Moreover, glomerular damage caused by autodigestion induces the release of cytokines and the production of oxygen-free radicals (31). Animal studies have shown that hypovolemia is essential in the early phases of AP and leads to AKI (32). In comparison with the control group, the glomerular filtration rate (GFR) decreased by 40% and the plasma volume by 26% four hours after the initiation of pancreatitis. Plasma infusion successfully reversed this decline in the GFR (32). Substances released by the necrotic pancreas, such as trypsin, chymotrypsin, bradykinin, histamine, prostaglandin E, endotoxins, and bacteria, have also been shown to be associated with AKI development (32). For example, histamine in pancreatic exudates may cause increased vascular permeability, hypovolemia, and hypotension (33). Peritoneal lavage increases urine output in patients with AP and AKI, suggesting that dialysis removes the substances responsible for causing AKI (33). Cytokines may be responsible for renal impairment. For instance, TNF-α can act directly on tubular and glomerular capillaries, causing tubular necrosis and ischemia. TNF-α can also cause endothelial cells to produce IL-1β, IL-8, and IL-6, which can cause thrombosis, renal ischemia, and the production of oxygen-free radicals (30). The urine N-acetyl-β-glucosidase to Cr ratio and early serum PLA2 activity are positively correlated in patients with AP, indicating that PLA2 may hydrolyze renal tubular epithelial cell membranes and result in AKI (33). Mucosal permeability is increased by inflammatory mediators that cause endotoxins and bacteria to move out of the colon. Endotoxins increase endothelin levels, which can induce tubular necrosis, reduced renal blood flow, and vasoconstriction, all of which can result in AKI (34). Ventricular compartment syndrome is an uncommon AP that can compress and impede renal blood flow in the venous and arterial veins. This compression can lead to increased renal pressure, reduced venous blood flow, decreased perfusion pressure, and increased venous pressure, all of which contribute to AKI (35). Hence, with the onset of AP, several pathophysiological pathways may result in AKI, which may increase blood Cr levels.

The primary protein in plasma, Alb, is produced by the liver and is crucial for preserving the osmolality of plasma colloids (36). Alb, a negative acute-phase reactant, is correlated with the degree of inflammation, prognosis, and mortality of several illnesses (4). Among its many biological activities, albumin may act as an antioxidant, prevent platelet activation and aggregation, and serve as a transporter for a variety of physiologically active substances (37). According to Shannon et al. (38), individuals with low Alb levels often have a poor prognosis. Serum Alb levels are typically low in critically ill patients with a variety of diseases. This decrease may be attributed to the role of Alb in increasing the production of several anti-inflammatory substances (such as lipoxins, hemolysins, and protective proteins) during oxidative stress to facilitate recovery from the disease (39). This process requires a significant amount of albumin. For instance, the Alb level has been shown to be a reliable indicator of death in older individuals with sepsis (40). Additionally, previous studies have shown that serum Alb levels may predict the prognosis of AP to some degree (12, 14). Nevertheless, in terms of forecasting short- and long-term all-cause mortality in AP, Alb performed worse than CAR, according to our results. Chronic inflammation or a patient’s nutritional state also influences Alb levels. Thus, utilizing Alb levels alone to predict the prognosis of AP has limitations. Therefore, we calculated the serum Cr: Alb ratio to predict the prognosis of patients with AP more accurately by decreasing the effect of individual variables on the regulatory systems via inverse changes generated by two separate processes.

The results of our study found that CAR was a stronger predictor of short- and long-term prognosis in AP compared with other control indicators. Alb and TCa are important indicators of a patient’s nutritional status and electrolyte balance. However, in AP, due to the inflammatory response and tissue damage, decreases in Alb and TCa may not be specific indicators because they are influenced by multiple factors, including the release of inflammatory mediators and an increase in endogenous damage molecules (41–43). Therefore, the use of Alb and TCa alone to predict mortality in patients with AP may be influenced by other factors, resulting in inadequate predictive power. The SOFA score, GCS and SIRS score are commonly used to assess the severity of disease and systemic inflammatory response in critically ill patients (8–11). However, these scores may have some limitations in predicting short- and long-term mortality in patients. For example, the SOFA score mainly focuses on the function of multiple organ systems and does not specifically address the pathological and physiological characteristics of pancreatitis itself, thus may lack sensitivity or specificity (4, 10). Similarly, although the GCS score and SIRS score reflect the patient’s level of consciousness and systemic inflammatory response, they may not fully consider the specific conditions of pancreatic tissue damage and inflammation in the prognosis assessment of pancreatitis (44, 45). CAR takes Cr and Alb levels into account and reflects a combination of renal function and nutritional status. In AP, elevated Cr levels may reflect renal dysfunction or systemic metabolic changes, while decreased Alb levels may indicate deterioration in nutritional status. Therefore, CAR, as a comprehensive indicator, can more comprehensively reflect the metabolism and nutritional status of patients, and is more closely related to the pathological and physiological characteristics of AP. Compared with other control indicators, CAR may have higher specificity and sensitivity and more accurately predict short- and long-term mortality in patients with AP.

Lactated Ringer’s solution is important in the management of patients with AP. Hypocalcemia is a common complication of AP and is closely related to impaired renal, pulmonary and cardiovascular functions (5). Lactated Ringer’s solution contains 3 mEq/l of calcium ions, which helps to increase serum calcium concentration and maintain electrolyte stability, thus preventing early organ failure (46). Wu et al. found that lactated Ringer’s solution reduces systemic inflammatory response and serum C-reactive protein in patients with AP better than normal saline (47). The systemic inflammatory response typically depletes large amounts of albumin, and Ringer’s lactate helps maintain albumin levels at normal levels (48). In addition, with the addition of sodium lactate to Ringer’s solution, lactic acid can be metabolized to bicarbonate in the body, enhancing the buffering effect in the body, which is important for metabolic acidosis associated with AP patients (49, 50).

The primary benefit of this study is its extensive real-world dataset, demonstrating that CAR can accurately predict the mortality of patients with AP across a wide range of periods. Nevertheless, this study has some limitations. First, our study was a single-center retrospective; therefore, multicenter prospective investigations will be needed to validate our results. To make our conclusions more reliable, we will conduct a multicenter study in China in the future. Second, the MIMIC-IV provided demographic information for our study, which included patients hospitalized from 2008 to 2019. However, such an extended timeframe does not ensure the consistency of treatment plans for patients with AP, considering the advancements in medical technology and therapeutic approaches. Consequently, it is plausible that the inconsistent treatment plans may have affected the findings of this study. Third, this study did not include participants under the age of 18 years; thus, further research is needed to determine whether the findings apply to people under that age. Additionally, due to the limitations of the database, we were unable to obtain the specific cause of death for each patient. This limitation prevented us from providing information on the impact of CAR on AP-specific mortality. Finally, the study cohort consisted of critically ill patients receiving ICU care; therefore, patients with typical mild pancreatitis may not have benefitted from the results.

## 5 Conclusion

In patients with AP, CAR may serve as an independent predictor of both short- and long-term all-cause mortality. The findings from our study may equip healthcare providers with additional tools for promptly diagnosing patients with AP, assessing disease severity, and treating individuals with poor prognoses. To support the prognostic utility of CAR in patients with AP, large-sample multicenter prospective studies are required.

## Data availability statement

The original contributions presented in the study are included in the article/supplementary material. Further inquiries can be directed to the corresponding authors.

## Author contributions

JW: Writing – review & editing, Writing – original draft, Resources, Methodology, Funding acquisition, Formal analysis, Data curation, Conceptualization. HaL: Writing – original draft, Methodology, Data curation, Conceptualization. HWL: Writing – original draft, Project administration, Data curation, Conceptualization. RS: Writing – review & editing, Software, Investigation. SC: Writing – review & editing, Methodology, Formal analysis. JH: Writing – review & editing, Investigation, Funding acquisition. HuL: Writing – review & editing, Resources, Project administration, Methodology. PY: Writing – review & editing, Resources, Investigation, Conceptualization. XC: Writing – original draft, Methodology, Formal analysis. YW: Writing – review & editing, Funding acquisition, Data curation. XZ: Writing – review & editing, Resources, Project administration. DW: Writing – review & editing, Supervision, Resources, Project administration, Funding acquisition, Formal analysis.
